# Supplementary figures and images for: Analysis of Whole Genome Resequencing Datasets from a Worldwide Sample of Sheep Breeds to Identify Potential Causal Mutations Influencing Milk Composition Traits
Source: Animals (Basel). 2020 Sep 1;10(9):1542. doi: 10.3390/ani10091542 (PMC7552124; doi:10.3390/ani10091542)

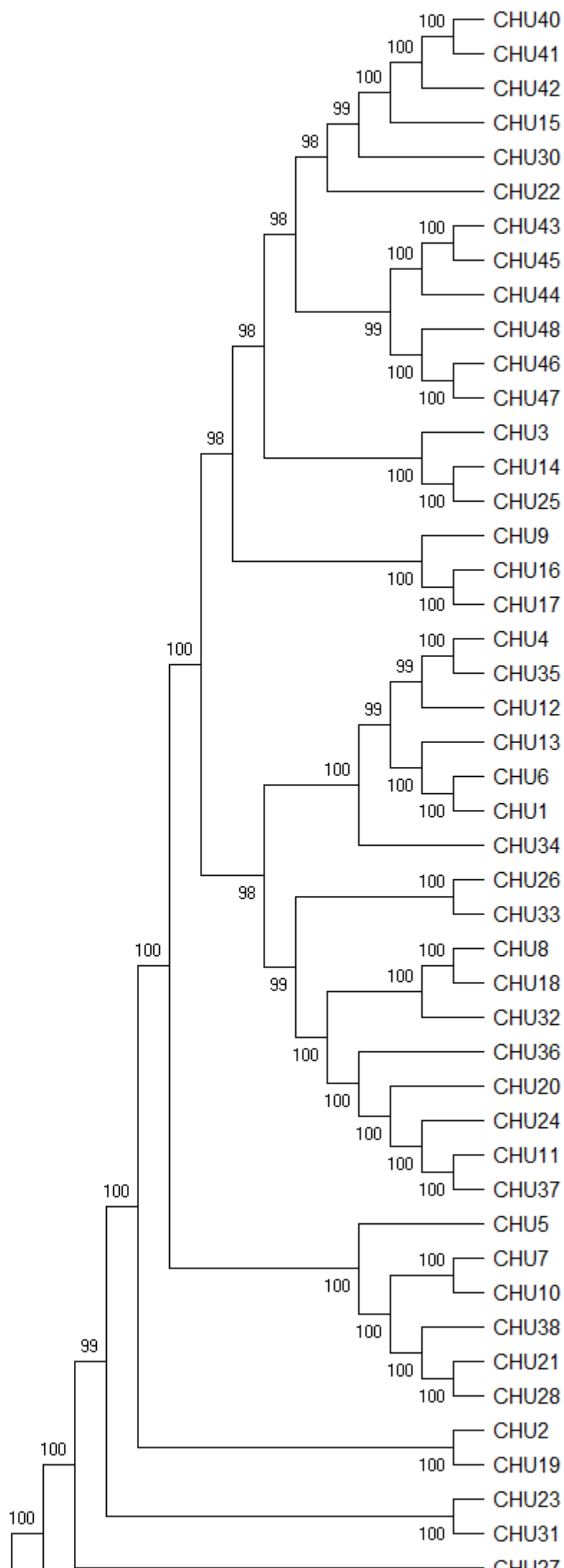

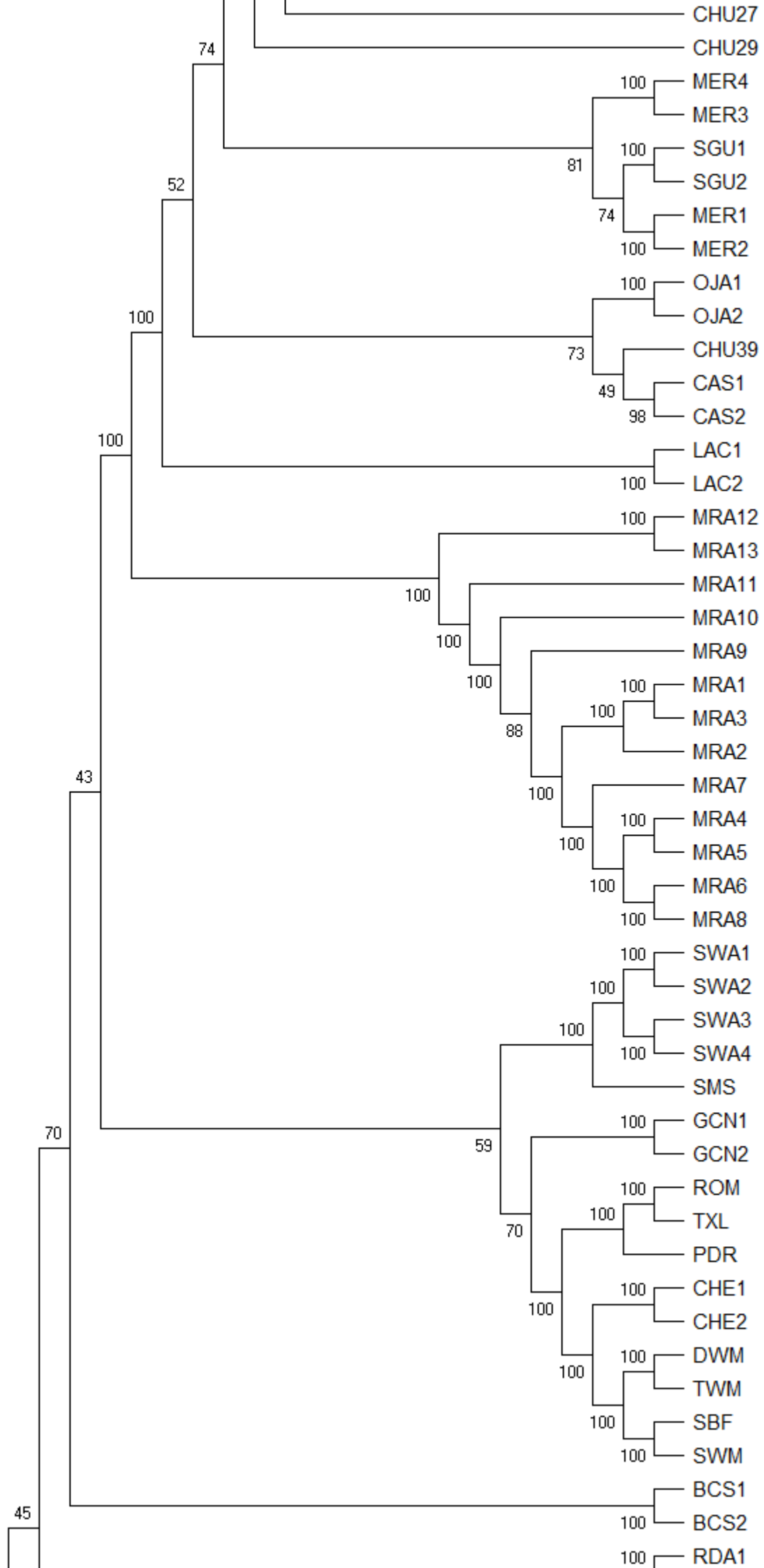

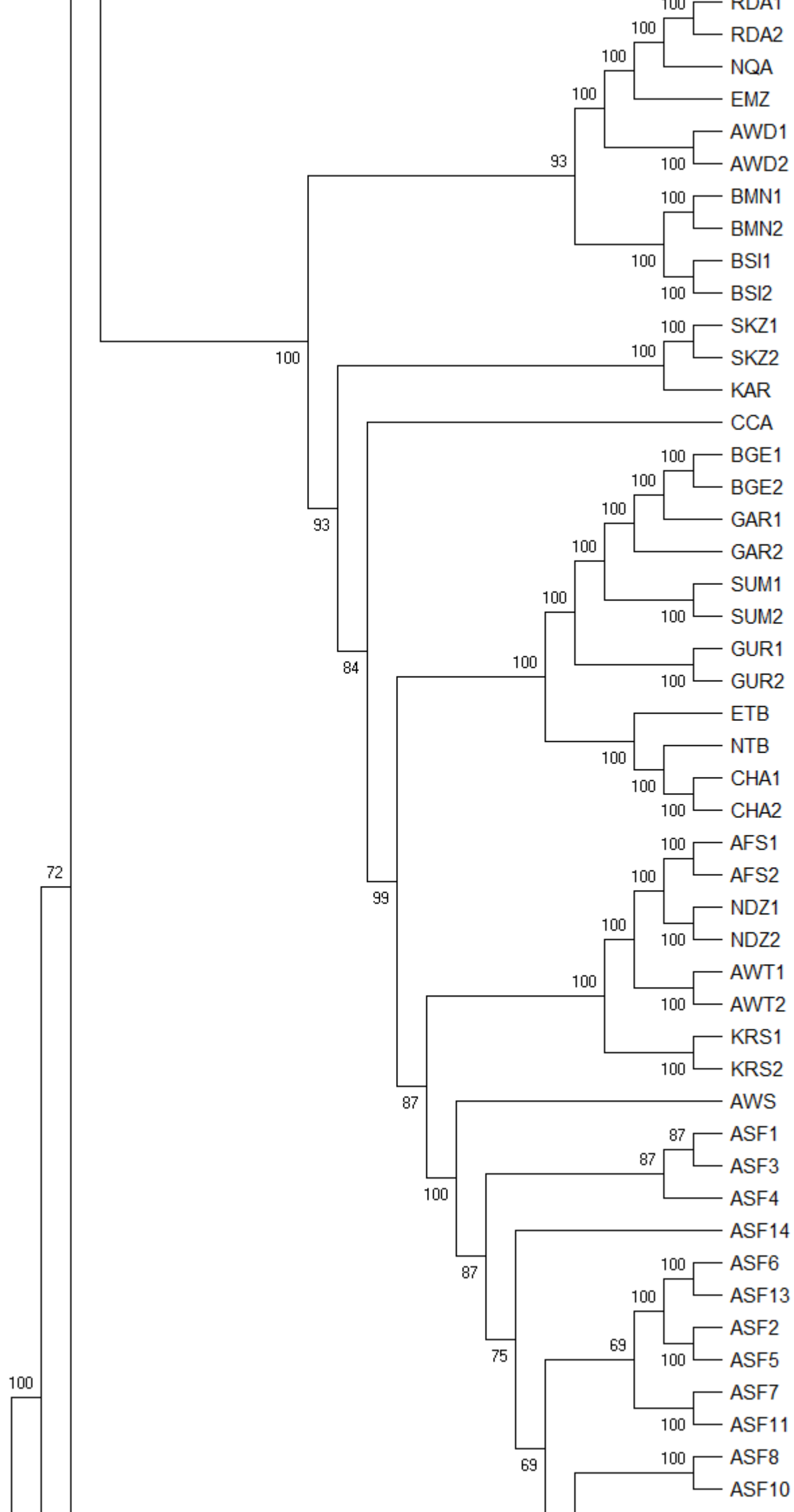

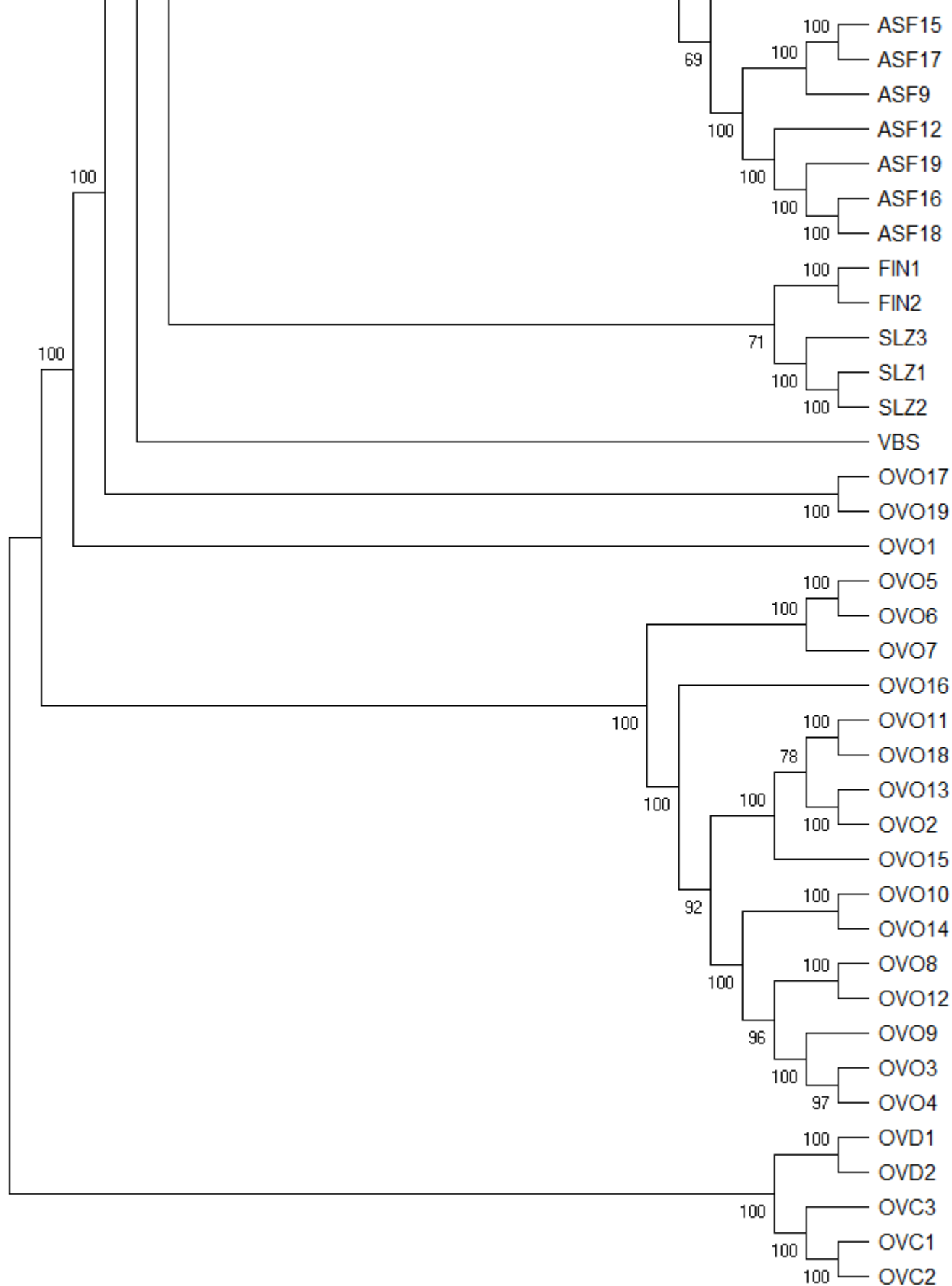

Supplement: Supplementary file 1 [file animals-10-01542-s001.zip › Figure S1.pdf]

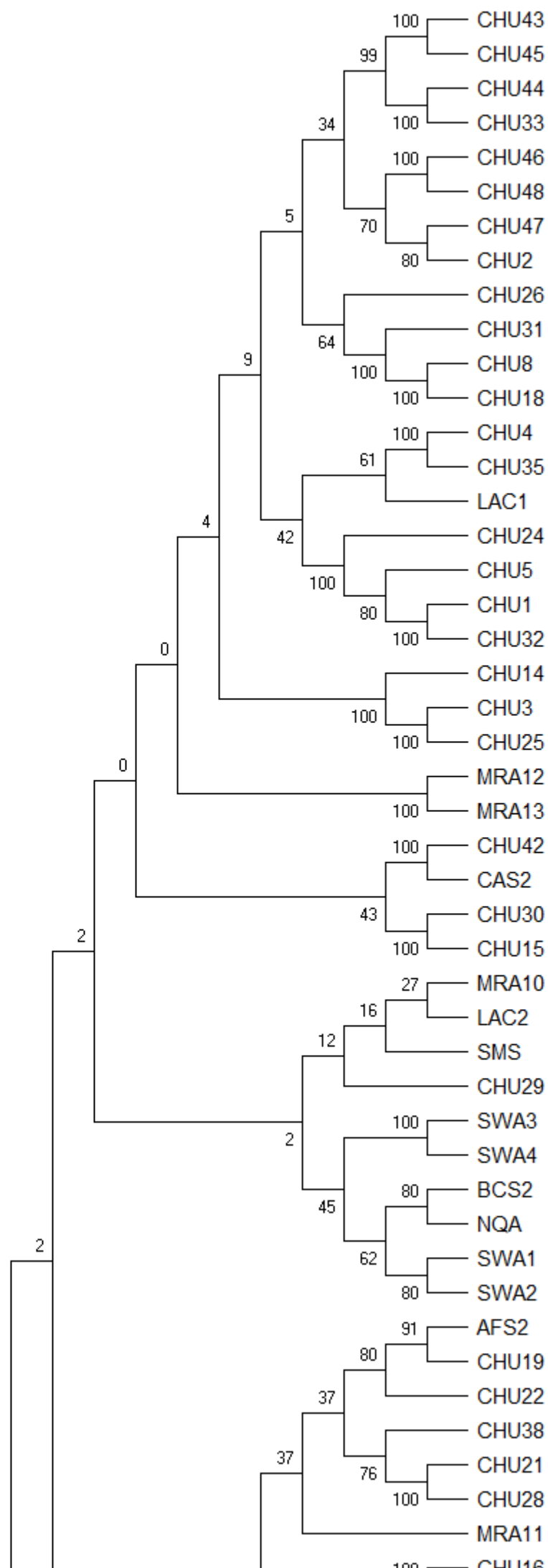

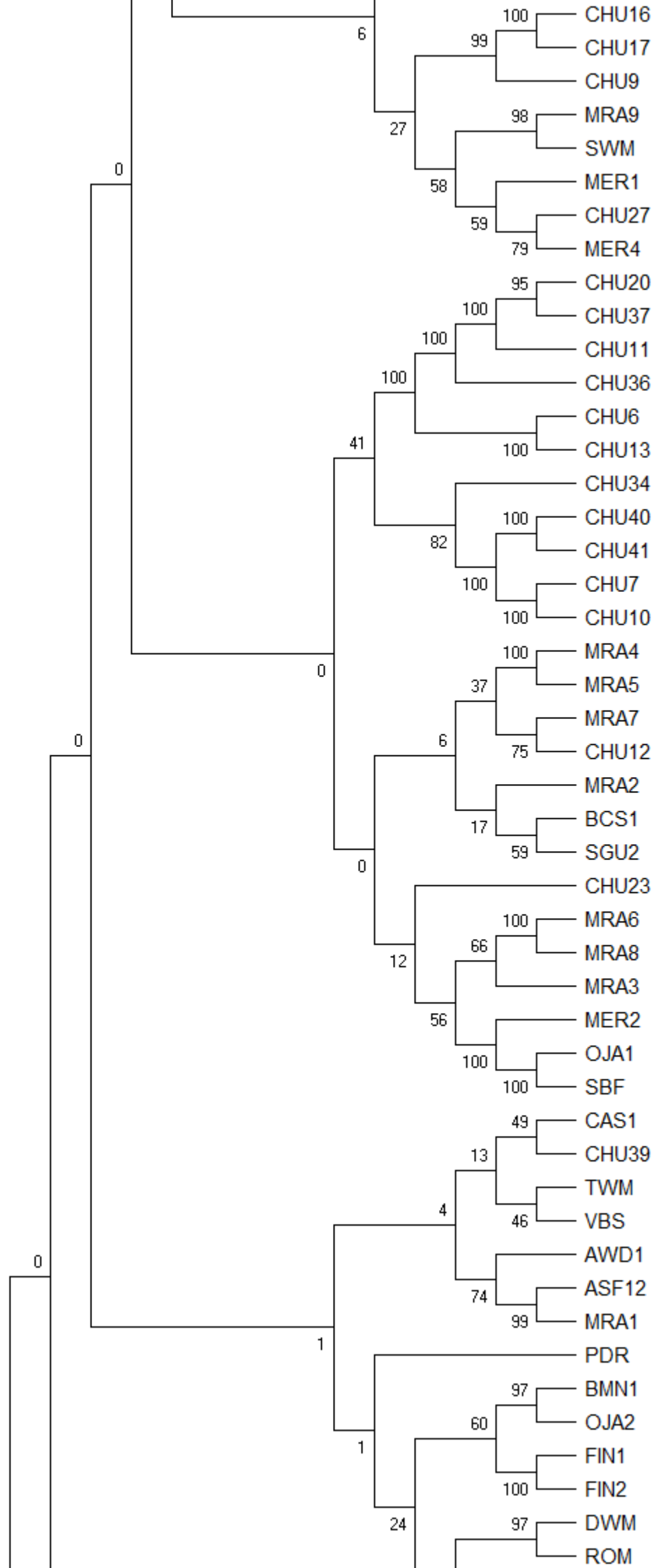

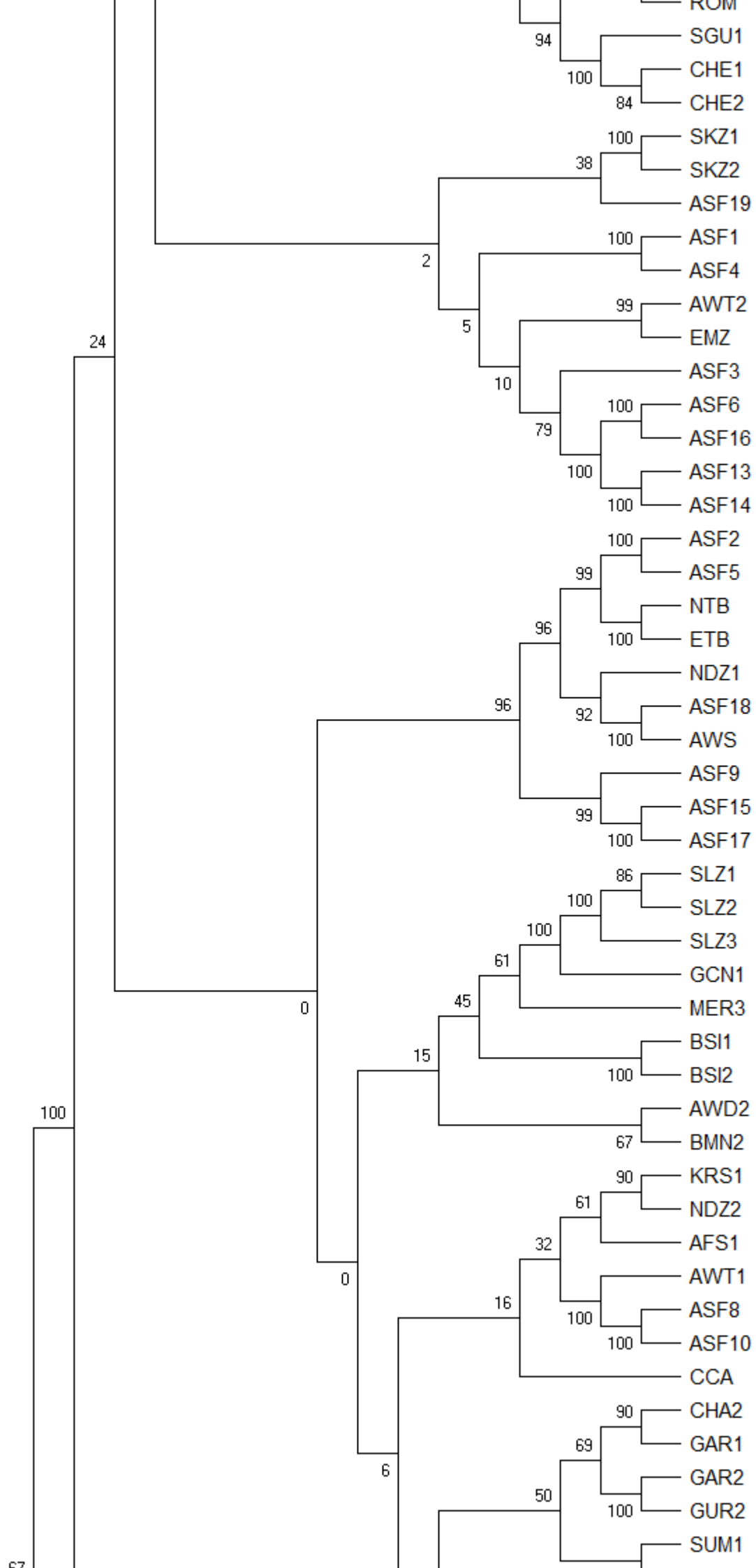

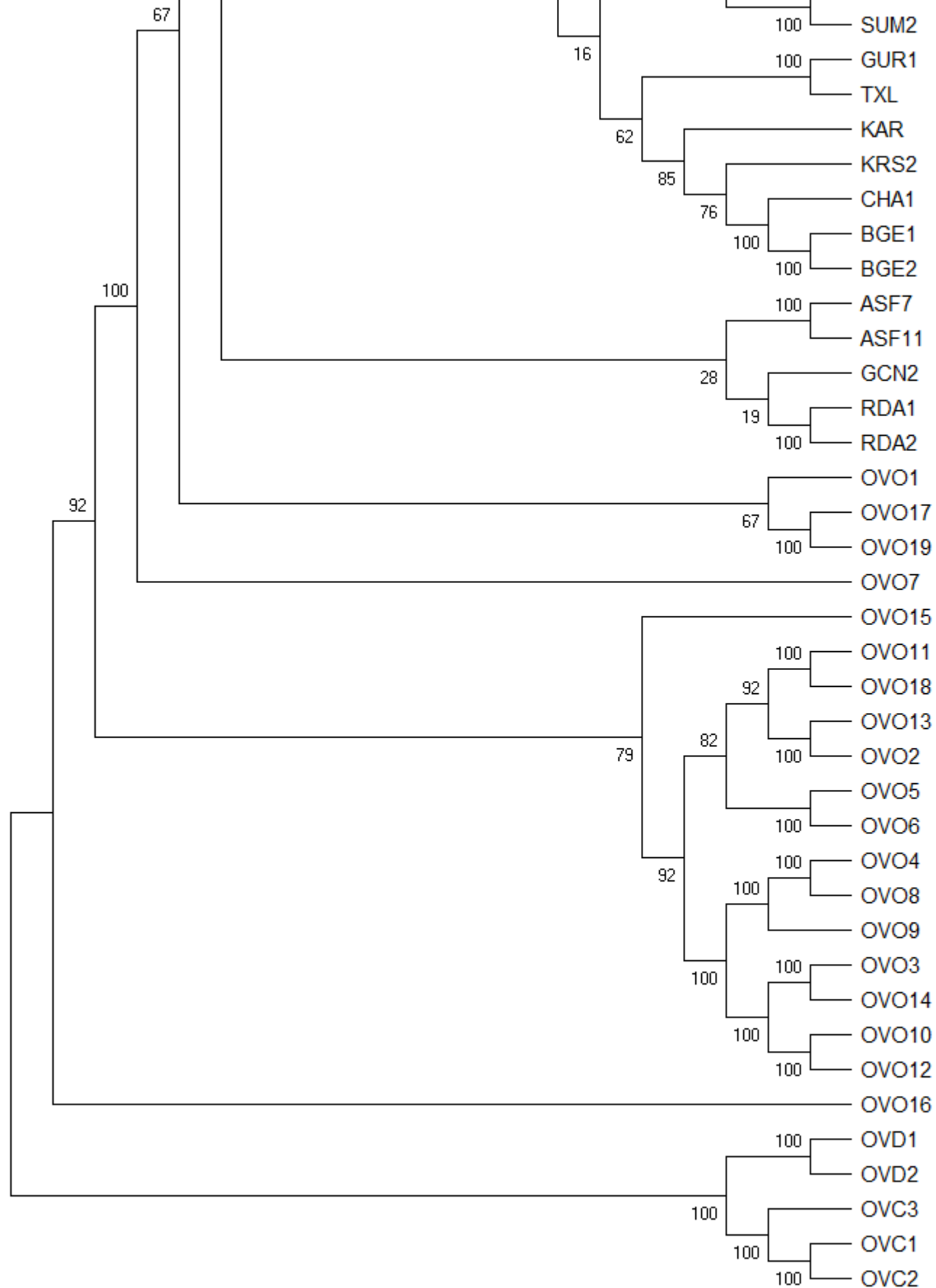

Supplement: Supplementary file 1 [file animals-10-01542-s001.zip › Figure S2.pdf]
